# “Thought provoking”, “interactive”, and “more like a peer talk”: Testing the deliberative interview style in Germany

**DOI:** 10.1016/j.ssmqr.2021.100007

**Published:** 2021-12

**Authors:** Astrid Berner-Rodoreda, Till Bärnighausen, Nir Eyal, Malabika Sarker, Puspita Hossain, Melkizedeck Leshabari, Emmy Metta, Elia Mmbaga, Daniel Wikler, Shannon A. McMahon

**Affiliations:** a Heidelberg Institute of Global Health, Heidelberg University, Germany; b Harvard T.H. Chan School of Public Health, Department of Global Health and Population, Boston, USA; c Department of Health, Behavior, Society and Policy, Rutgers University, Piscataway, USA; d Center for Population-Level Bioethics, New Brunswick, USA; e BRAC James P Grant School of Public Health, BRAC University, Dhaka, Bangladesh; f McMaster University, Hamilton, Canada; g School of Public Health and Social Sciences, Muhimbili University of Health and Allied Sciences, Dar es Salaam, Tanzania; h Social and Behavioral Interventions, Johns Hopkins Bloomberg School of Public Health, Baltimore, USA

**Keywords:** Interviews, Qualitative, Methods, Deliberative, Ethics

## Abstract

Proponents of the newly-developed “deliberative interview” argue that examining complex issues requires more dynamic and engaging interview exchanges. Unlike traditional qualitative interviews, deliberative interviewing champions opinion sharing, active debates and similar speaking times by both sides throughout the interview. Drawing on 20 interviews with health experts in Germany, we examined the process and outcome of deliberative versus conventional interviews on the topic of informed consent. The deliberative interview expedited clarity on the issue, led to more nuanced discussion and generated more knowledge overall, but was challenging because it broke the mold for traditional interviewing. Alignment in terms of gender, age, personality and professional background facilitated rapport, regardless of interview style. To manage expectations, we recommend a thorough, perhaps video-based explanation of the deliberative style prior to the interview. Deliberative interviews can bolster knowledge generation for complex issues and can be applied in public health and beyond.

## 1 Introduction

The concept of deliberation can be traced back to the writings of Socrates and Aristotle (Gundersen, A. G., 2000; Shefali, 2009) and has thus been used for over 2400 years. In recent decades, deliberative approaches have become popular in academic fields including political science (J. Fishkin et al., 2000; J. S. Fishkin & Luskin, 2005; McCoy & Scully, 2002), environmental studies (McCrum et al., 2009; Palomo et al., 2013) and health (Abelson et al., 2013; Braunack-Mayer et al., 2010; Degeling et al., 2018; Dolan et al., 1999; Rychetnik et al., 2013).

The term “deliberation”, however, means different things to different people: “there are as many definitions of deliberation as there are theorists” (Mutz, 2008, p. 525). While there seems to be widespread agreement that “deliberation” involves the weighing of options, reasons or considerations (Abelson et al., 2003; J. S. Fishkin & Luskin, 2005, p. 285; Mansbridge, 2015), political theorists disagree on whether deliberation should aim for consensus or for respecting the position of other participants; on whether it should be conducted publicly, promote public engagement and inform public policy or can apply to private dialogues; and on whether power may counteract equality and persuasion (Abelson et al., 2003; Burchardt, 2014; Dryzek, 2002; J. S. Fishkin & Luskin, 2005; Mansbridge, 2015; Mutz, 2008; Thompson, 2008).

Deliberative approaches are commonly employed in group settings and can take various forms (Scottish Government, 2009). Building on Curato’s suggestion to include deliberative elements in the interview situation (Curato, 2012), a new qualitative interview style, the “deliberative interview” has been proposed (Berner-Rodoreda et al., 2020). It is preceded by a background briefing on the interview theme and is described as a reasoning together of interviewer and interview partner (IP) with the aim of generating solutions. The authors characterized the deliberative interview by similar speaking time for interviewer and IP, lesser importance of rapport and an egalitarian relationship between interviewer and IP to the extent that roles could be exchanged. Interview partners would be encouraged to ask questions and the interviewer could share knowledge and experience (Berner-Rodoreda et al., 2020).

Our study applied Mansbridge’s definition of “deliberation” as “mutual communication that involves weighing and reﬂecting on preferences, values and interests regarding matters of common concern” (Mansbridge, Jane, 2015, p. 28) with the aim to explore options and positions and gain new insights and knowledge for our study subject of “informed consent”. Mutual respect was of greater importance to us than reaching consensus in the interview situation. We tested and compared the novel “deliberative” interview style with a conventional qualitative interview in terms of comparing the interview process and knowledge generation. To our knowledge, this is the first comparison of its kind; in testing and applying a new interview style, we aim to widen research options for qualitative researchers and to extend the tools for generating compelling epistemic data by ascertaining the IP’s position through dialoging and interrogating and building on each other’s positions.

## 2 Methods

### 2.1 Study design

This qualitative research is based on in-depth semi-structured interviews with health experts. The interviewer (and lead author) conducted face-to-face interviews in Baden-Württemberg, a state in southern Germany. Half of the interviews were deliberative interviews in which both IP and interviewer would share information and reason together, the other half were conventional qualitative interviews in which the interviewer kept a neutral stance – the latter interview style also included questions about the interviewee’s own experiences and personal reactions to different scenarios as well as questions aimed at the interviewee reasoning about the subject matter.

This qualitative multi-country study is part of an international research project on establishing an ethics framework for health policy trials. An article on participants’ understanding of informed consent requirements across the international research project is currently under review. This paper, by contrast, focuses on the experiences within Germany specifically, with a methodological comparison of the two interview styles. Analysis in other countries is underway. The research team held weekly debriefings to share interviewing experiences, content insights, and to inform the interviewing process (McMahon and Winch, 2018).

### 2.2 Sampling, data collection and data analysis

We selected IPs purposively from the following five sub-groups of health experts: researchers, medical doctors, policymakers, representatives of non-governmental organizations (NGOs) and ethicists or members of ethics commissions. After identifying key interview partners for each sub-group we used snowball sampling to identify others (Patton, 1990). All participants provided written consent prior to being interviewed. Data collection ceased when saturation was reached (Morse, 2000).

We matched two potential interview partners in the same sub-group of health experts and randomized using an online random generator (https://www.matheretter.de/rechner/zufallsgenerator). If the random generator selected “A” for a paired group of medical doctors, a conventional interview would be conducted with the first health expert in the paired group, a deliberative interview with the second expert. If “B” was selected, we would first conduct a deliberative interview. After piloting interviews with health experts from November 2018 to January 2019 and discussing findings in the international team, the research team adapted the interview guide further. Interviews in Germany were conducted between February and April 2019 (n ​= ​20) by the first author.

IPs were usually contacted per phone or email, informed about the research project and asked if they were willing to participate in this study. A few days prior to a deliberative interview, the interview partner would be asked for a “dialogue” or “conversation” rather than an interview and told that the dialogue will be based on concrete examples; s/he was also sent a one-page background information about the interview topic (see Supplementary File 1). The interview partner of a conventional interview, by contrast, would not receive any detailed information prior to the interview. Interviews were conducted at a convenient place for the IP (mostly workplace or home), lasted 70 ​min on average and were mostly conducted in German; interviews with international health experts living in Germany were conducted in English. All interviews were audio-recorded, transcribed and analyzed using NVivo Pro 12. In debriefings, the research team agreed on the categories for comparing and analyzing the two interview styles.

Codes were discussed and drawn up jointly by the international team; they were partly drawn from an overview article on interview styles (Berner-Rodoreda et al., 2020) and partly from debriefing notes. The codebook was further adapted during weekly debriefing sessions wherein interview experience and findings were discussed. Codes related to the topics discussed, such as “health policy experiments”, and solutions such as “referendum”, “decision by government”, or “consultation with experts”; codes were also applied to reflect agreement and disagreement as well as a change of view, speaking time, and feedback to the interview style. As new codes were added by the team, transcripts were re-coded (Author A and others, under review). Interviews were analyzed and contrasted in terms of characteristics of deliberative interviews (Berner-Rodoreda et al., 2020) and the experience of conducting the interview (Supplementary File 3) and in terms of knowledge generation (Supplementary File 4).

### 2.3 Interview guides

Interview guides covered the topic of informed consent in the context of health policies, health policy trials, clinical trials and medical interventions. Our particular interest was in consent requirements for the testing of health policies: would this be a political decision which did not need consent, would it need to be approved by an ethics council or commission, would it need consent by the entire population, i.e. a referendum? In order to explore the position of interview partners, the interview guide for both interview styles provided concrete examples of the first three contexts – health policies, health policy trials and clinical trials. While the conventional interview guide only contained a few potential probing questions after the main question or scenario, the deliberative interview guide additionally contained reminders to share one’s opinion and experiences and information for challenging the interview partner (see Interview Guide in Supplementary File 2). In order to demonstrate how the engagement of the interviewer differed between a conventional and a deliberative interview, we provide below a concrete example and probing questions. The example draws from a hypothetical case wherein a government wants to learn if by screening the entire population for a sexually transmitted infection (syphilis) more cases could be detected and treated. The study design is based on randomizing all health facilities in the country: the active arm offers screening to all patients – the individual patient can, however, opt out. The control arm continues with business as usual: testing is only done if requested by the patient or if the patient shows symptoms. In both interview styles the IP was asked how s/he viewed this government approach and the alternative approach of asking local leaders or councils to decide on the participation of the health facilities in their area. In the deliberative interview guide, possible interviewer reactions were listed to counter the IP’s position, see Box 1.

Box 1 **Interview guide excerpt for deliberative interview**

Arguments against the alternative approach of asking local leaders/councils, if IP is for it:

I personally do not really think it would be necessary to go through the local leaders. I think, if health facilities are randomized in the whole country, there is no bias in the distribution of where the screening is offered and where it is not offered. From that point of view I regard government randomization as better science than doing it through local leaders and councils, as their personal interest or apprehension about this trial might confound the results. It is not randomly distributed – e.g. local leaders may refuse the intervention precisely at precincts that (as the leaders suspect) are those where the intervention is likely to fail, as people may be embarrassed to be tested for syphilis and therefore not make use of the health facilities. What’s important to me is that a trial is conducted before a new policy is introduced. What is your view on this?

Arguments for the alternative approach of asking local leaders/councils, if IP is against it:

At the same time, having local leaders engaged might also encourage people to go for testing and it is generally always good to consult with people beforehand. This way, if and when some individuals are offended by the offer of testing for syphilis, they could be answered that in a sense their representatives have offered their consent to this. While representatives’ consent is not precisely individuals’ consent, the latter is impracticable, and representative consent is the closest second best.

Alt-text: Box 1

As in all qualitative interviews the actual questions and the comments by the interviewer would depend on the answers of the interview partner and the dynamics of the interview exchange. The arguments provided were suggestions for a response by the interviewer; they were not prescriptive.

### 2.4 Positionality and reflexivity

In order to appreciate the interactions and power dynamics between interviewer and interview partners, I provide some background on myself as the interviewer and my views on the subject matter: I am a German middle-class and middle-aged woman with an academic background in social-anthropology, educated to master’s level and pursuing a PhD in Global Health. With more than 20 years of international NGO experience, many of them as a global advisor for HIV and gender issues and serving on boards of HIV and health-related advocacy networks, I have experience interacting with diverse stakeholders. I presumed that my age would lend itself to engaging with younger and older interview partners alike, and that my life and work experience may offset any power imbalance with interview partners of higher education or position.

I had acquainted myself with the literature on ethical issues of health policy experiments and in particular consent issues – the subject matter of the interviews. It was clear that the deliberative interview would require engagement from both the interviewer and the interview partner as both participate fully in the dialogue. This also raised the question to what extent one may counter an argument for the sake of debate. As the interviewer and discussant, I still largely wanted to be true to myself. I would sometimes challenge a position that I myself shared but by and large I presented my own personal views and experiences. The suggestions for reactions for the deliberative interview presented above had been agreed upon in the team and also largely represented my own opinions. Having researched various interview styles, I was curious how an interview would work in which I could fully participate. The interview topic of informed consent touched on broader issues of group consent and representative consent. Having been socialized in a democratic country and having witnessed the havoc of the Brexit debates, I believe in the benefits of representative democracy and have become more skeptical about the benefits of conducting a referendum on controversial issues. For the central question at stake in our interviews: if health policy trials – the testing of health policies by a government – should be treated like health policies or clinical trials in terms of consent, I leaned towards seeing them as health policies which democratic structures would have to decide upon based on evidence and expert knowledge. But I was also aware that I regarded certain examples as more risky for the individual and could therefore also relate to opinions that emphasized individual consent over democratic decisions.

## 3 Results

The 20 participants interviewed ranged in age from 26 to 75 years with a majority older than 40 years . Half of all IPs were women; IPs above the age of 60 were male and held a doctorate (see Table 1).

**Table 1:** Sociographic data of interview partners.

	Conventional (n) 10	Deliberative (n) 10	Total (n) 20
Gender			
Male	6	4	**10**
Female	4	6	**10**
**Age**			
up to 30	1	1	**2**
31–40	3	2	**5**
41–50	1	2	**3**
51–60	1	4	**5**
61+	4	1	**5**
**Education**			
up to Bachelor's Degree	1	2	**3**
up to Master's Degree	2	2	**4**
up to PhD	7	6	**13**
**Health Experts**			
Medical	2	2	**4**
Policymakers	1	1	**2**
NGO representatives	2	2	**4**
Ethicists	2	2	**4**
Researchers	3	3	**6**

In both interview styles, substantive knowledge around informed consent was generated: in the deliberative style knowledge was co-constructed through dialogue, in the conventional qualitative style the IP mostly self-deliberated (detailed below). While some IPs in both interview styles changed their opinions, agreements and disagreements between interviewer and IP were characteristics of a deliberative interview as the interviewer remained neutral in the conventional interview style. Average speaking time varied between interviewer and IP in the two interview styles and tended to be more equal in the deliberative style, yet an equal relationship between interviewer and IP that allowed a complete change of roles was harder to achieve. Both IP and interviewer enjoyed and regarded the deliberative style as easier for complex interview topics. We first present main findings regarding the characteristics of the interview styles (a detailed comparison of deliberative and interview styles is presented in Supplementary File 3) and will then examine the knowledge generation of both styles. All excerpts provided refer to specific health experts numbered chronologically according to the timing of the interview within each interview style.

### 3.1 Joint deliberative versus self-deliberation

The deliberative interview allowed both IP and interviewer to reason together to advance knowledge by building on and responding to each other’s arguments; in conventional interviews the IP had to rely on his/her own knowledge to generate answers and solutions. The following quotes show a deliberative debate contrasting with the self-deliberation that was characteristic of conventional interviews (see second quote). The first excerpt is based on the example of trialing water fluoridation in cities before introducing it as a health policy, the second on randomizing health facilities for trialing routine syphilis screening.

**Deliberative interview with ethicist**

I: …. In one city you would offer fluoridated water but not in the other. In order to compare that, who would you ask for consent?

IP: GBA (Federal Joint Committee of medical doctors, hospitals and insurance funds in Germany)

(…)

I: How about a city council or

IP: But who? They would not have a clue.

I: The city council with experts.

IP: No, no!

I: You would be against that? But these are our elected representatives.

IP: Yes, but they are not health experts.

I: True but one could bring them together with experts – experts who are for and against the intervention and then the elected representatives could make an informed decision. How do you see that?

IP: I don’t think that they can do that.

I: You think they have too little health expertise?

IP: Yes, at communal level - no. They can be consulted but they can’t gauge the interests behind this.

I: But if I want to test this, I will need the buy-in from various cities. I don’t think you are able to impose this on cities to say: “Okay, in your city this will be offered.” This needs

IP: They can consent but the decision has to be made elsewhere.

I: The general decision about the experiment (health policy trial) has to be taken at a different level but the decision in which cities it should be tested should involve decision-makers at city level in my opinion.

IP: But what do you do, if you have a regional CSU (Bavarian conservative party) councilor in Bavaria who is a board member of GSK (pharma company Glaxo-Smith-Kline)? Are they the right ones? I don’t think so.

I: I still think you have the full spectrum of opinion at city or regional council level. You have the various parties represented. They will reflect the opinions in the general population, and concerns would be verbalized when a city or regional council holds a debate about this. For me this would be a decision-maker that is elected and politically legitimized.

IP: Yes, but still has no expertise in that matter.

I: I think the expertise has to come from the outside. Experts need to be invited so that councilors can make an informed decision or do you see this differently?

IP: I don’t see how they should be able to make an informed decision. I have my doubts that this is possible. If it were possible, I think it would be good. But this is the subjunctive. (ethicist 1)

**Conventional qualitative interview with researcher**

I: Alternatively, one could have asked local councils, if the facilities in their ownership take part in this study. How do you view this approach?

IP: Yes, that was what I said before, that one could also involve providers of health facilities or the facilities. That would certainly make sense against the backdrop of having greater compliance in those who want to participate. That needs to be weighed-up. You have a selection of the facilities on the one hand, since some do not want to participate. Perhaps these are in the regions where the problem is greatest; that would be possible. That means one would limit the generalizability of the results and perhaps one may have a smaller benefit in the end… On the other hand, if one only includes those (facilities), which are willing to participate, one will definitely have higher compliance of participating facilities because they get support from the providers. That means that a more rigorous evaluation may be possible because both groups are compliant in being randomized into a particular group or for a particular intervention. That is a difficult consideration. It is also difficult to say how one would weigh this up and what the best approach would be. It certainly depends on the country context and on previous experience of implementing those studies. (researcher 3)

The first interview excerpt shows that IP and interviewer hold different opinions and contradict each other about who would need to consent for the health policy trial (HPT) to be conducted. While there was agreement that the overall HPT would need to be approved and that it needed the involvement of health experts, if and how local consent for conducting the HPT should be obtained was not fully agreed upon – the IP highlighting the danger of the pharma industry influencing decisions, the interviewer upholding the role of elected councilors who could draw on expert opinion. While the IP conceded that the suggested procedure by the interviewer may work in theory, she expressed doubts about it in practice underscoring issues of conflicts of interest and lack of expertise of local decision-makers thus adding further nuance to the dialogue.

In the second interview excerpt, the IP skillfully presented arguments for and against involving local councils for randomizing health facilities. We noted that many IPs, especially those well-informed on the subject matter tended to self-deliberate advantages and disadvantages, others shared their initial reactions or thoughts.

### 3.2 Relationship equality between interviewer and IP

The deliberative interview style has been described as a discourse among “equal partners” (Berner-Rodoreda et al., 2020). Equality was almost achieved through speaking time, which was more evenly balanced between IP and interviewer in the deliberative interview (52% versus 48%). Yet it could vary – in some deliberative interviews speaking time of the IP could take up close to 70% and thus resemble a conventional interview. This was mainly the case when the IP shared a lot of examples. When the IP tended to give short answers or felt unsure about his/her opinion, the interviewer dominated over the IP. In terms of interrogating and challenging each other, the deliberative interview showed more equality than a conventional interview but only rarely reached the level of fully exchanging roles between interviewer and IP.

### 3.3 Change of mind

In both interview styles we saw a change of opinion in IPs through the interviewer challenging, pointing out inconsistencies or because the IP regarded one example as riskier than another. The following excerpts show the changes of opinion in the conventional and the deliberative interview going in opposite directions. In the deliberative interview the IP felt that institutions should be asked for consent and then agreed that it would bias the results of the study. In the conventional interview, the IP first argued that randomization itself is a political decision, then expressed second thoughts about it adding arguments for why it may make sense to ask implementers for prior consent. The example refers to the national randomization health facilities to test a syphilis screening approach before possibly introducing it as a health policy.

**Deliberative interview with NGO representative**

IP: I would think it is sensible to involve the institutions which are tasked to implement this, to ask for their consent, yes and also the patients. That would be for me …

I: I would view this differently. I see your point that if I oblige somebody to implement measures, and the institution does not fully support it, it may have implications for the implementation. On the other hand, if I ask each institution, whether they would like to participate, it will create a certain bias in my opinion, because we might have a major problem [with syphilis] in those areas where the institutions are not willing to implement it. From a scientific point of view, I would say, it is not really appropriate to ask each institution, if they would like to participate or not; in terms of the motivation of implementers, on the other hand, it may have advantages.

IP: Yes, I understand. I guess the question is probably again… what type of involvement in the planning of such a policy or a policy experiment is regarded meaningful? And there I would say for this example, to ask the institutions or the selection of institutions: what would you regard as practicable; based on your practical experience: who would you involve? Or how would you implement it at a low threshold? But yes, this is not about consent. No, I agree with your arguments.

I: So would you say, a consultation, yes, but then it is a government decision, in which arm of the study each institution is placed? Or would you see this differently?

IP: No, no, this needs to be a government decision; otherwise the results will be biased; I agree with that. (NGO 2)

**Conventional qualitative interview with researcher**

I: The government could have also decided to approach the local councils and ask them if the hospitals or clinics in their area would take part in this study. How do you view this approach?

IP: This is important, because… it also depends, because if the government implements this and doesn’t inform the facilities and the facilities cannot afford … either because they have a shortage of personnel or, I don’t know, something in the system that will not allow a smooth procedure for this syphilis testing, then it’s important to inform the hospitals and stakeholders and the area.

I: To inform or to ask them?

IP: To ask them, because if they don’t understand why this is done and if they can’t take part for other reasons, like I said, shortage of nurses or maybe their laboratories cannot handle testing for syphilis, I don’t know, then that will also affect the results of their intervention.

I: Before you said there would be less of a bias if it was just randomized by the government. Are you changing your opinion?

IP: You are right. It’s not changing but you made me think about another area, when you asked me again. The problem is, because… if you don’t inform them and you force them to take part and then they have some hurdles that they cannot overcome to make this happen…then… it won’t help the government. You know what I mean? But you have to also watch here for other issues like corruption and people who will take advantage. Maybe if the government is allocating money for those institutions to be able to handle this research for this intervention and maybe you will get corruptions and some of the…That’s my fear when I say they shouldn’t inform and just do it.. I see also the other point where you should inform, just to avoid resistance or false results. (researcher 2)

### 3.4 Agreement and disagreement

Agreement and disagreement were prerogatives of a deliberative interview as the interviewer remained neutral in the conventional qualitative interview.

While IPs disagreed or challenged the interviewer in six out of ten deliberative interviews, the interviewer disagreed with and challenged IPs more than vice versa. Agreements exceeded disagreements in all deliberative interviews and thus mirrored an ordinary conversation. Agreement went both ways – from IP to interviewer and from interviewer to IP. The discussion below explored the possibility of asking everyone for their consent by holding a referendum on whether to test syphilis screening before introducing it as a policy. Analogous to an ordinary conversation, one idea built on another.

**Deliberative interview with medical doctor**

IP: If this was about something else that I would not know much about, environmental issues or something like that, well, if I can’t say much on the issue, then I think it is frankly speaking superfluous to ask me about it.

I: Yes, and I think sexually transmitted diseases are a delicate topic for many. I am not so sure that I would ask the whole population about it. I can’t imagine that this would lead anywhere.

IP: Perhaps moral and religious ideas also play a role in that regard.

I: Exactly and that may lead to many being opposed to it because they find it embarrassing or

IP: Right and then it will become more difficult. Okay, if it is a topic which is not loaded socially or religious-morally, then I might be able to say, okay, there is information, but

I: Yes, I feel the same way but I also think, Brexit has shown us plainly that people often do not fully understand what they vote on. I think a referendum can be an important instrument but not for everything.

IP: I would see it the same way. (medical doctor 1)

The frank discussion or dialogue in the deliberative interview also facilitated exploring and clarifying the IP’s position on the theme, which was harder and often almost impossible to achieve in a conventional interview.

### 3.5 Rapport

In the deliberative interview the interviewer and IP had to engage and debate while maintaining respect and collegiality. Since the deliberative interview mirrored a dialogue or ordinary conversation rather than a formal interview, one had to be comfortable in the other’s presence, and a joint laugh was often an integral part of the conversation; a smile might accompany playing devil’s advocate. Rapport therefore seemed to have higher relevance for the deliberative interview as this IP suggested:

**Deliberative interview with researcher**

I think, if there isn’t some kind.. of connection or fluidity between the interviewer and the interviewee, if it’s this interactive, then it can get difficult. If I didn’t feel comfortable or if I felt awkward, I think, it would be even harder for me, if it felt like a conversation with somebody that maybe I wasn’t comfortable having a conversation with. It does feel a little more personal this way. And if I feel some positivity and connection too, then it actually increases my willingness to talk. But I think, if I didn’t, then it’d actually decrease my willingness to talk, because it really feels personal. (researcher 1)

In short establishing and maintaining a good atmosphere, which is an important condition for all interviews, seemed to be particularly relevant for the deliberative interview style. Factors such as age, gender, professional background, and personality also played a role in establishing a connection for dialoguing and deliberating together, with similarities between interviewer and interview partner facilitating the dialogue.

### 3.6 Feedback and assessment of interview styles

Both IPs and interviewer found it difficult to step out of the conventional interview roles in the deliberative interview. While IPs asked a question in all deliberative interviews (and most conventional interviews) - mostly to clarify an example further or to provide an idea for a health policy - only in three deliberative interviews did the IP ask for the interviewer’s opinion.

IPs perceived the deliberative interview style as unusual and surprising in hearing the interviewer share an own opinion, yet viewed it as more enjoyable; some commented favorably on the interview not solely depending on the IP’s answers and reflections but on building on each other’s arguments and developing ideas together.

**Deliberative interview with policymaker**

I found this dialogue with you very pleasant and I found that, I think, it’s good that you develop ideas through this dialogue. With issues like these which can be viewed very differently, where opinions may vary.. I found this a pleasant style of interview – practically like a dialogue. (policymaker 1)

IPs described the interview style as “thought-provoking”, “very exciting”, “like a peer talk” and “better than answering set questions on one’s own”.

IPs with whom a conventional interview was conducted commented on the subject matter as being “stimulating”, “interesting”, “not having thought about these issues before” and they described the difficulty of presenting a coherent opinion.

**Conventional qualitative interview with researcher**

but I think it’s a very difficult subject... And even me, it made me think now about the things, you know, I thought I had an opinion about, but now I am realizing I am actually, I myself I am grey, I don’t know, if it’s good or bad. We are good at judging after the things happened. But in the end, if you are a Minster of Health, it’s not an easy job to do the right thing. (researcher 2)

From the interviewer’s perspective the deliberative interview felt more relaxed as one could freely share one’s opinion as opposed to a conventional interview where one had to appear neutral. Both interview styles were pleasant and interesting to conduct with knowledgeable, experienced and engaged IPs irrespective of gender and age who expressed ideas well and added own examples. In the conventional interview style it was often challenging and difficult to determine the IP’s position – for some of these interviews, a deliberative approach may have worked better, yet if the IP in a deliberative interview talked profusely, it felt more like a conventional interview. When the IP was unsure about his/her position, the interviewer shared more and the IP tended to agree with the interviewer. The Interviewer’s style of letting IP generally finish train of thought may have established more rapport, yet may have also made interview less confrontational. Interviews were easy to conduct with IPs of same gender and younger or same age and with IPs with similar professional work, background or personality irrespective of gender. Willingness to engage with each other’s ideas seemed most crucial for co-generating knowledge.

### 3.7 Knowledge generated in and through the interview situation

In both interview styles the knowledge generated emerged as an integral part of the interview process, the interactions, questions asked and information shared. Whether the one page background briefing on the theme sent to IPs prior to conducting a deliberative interview made a difference in knowledge generation is difficult to gauge. The sharing of personal examples by the interviewer generated additional insights on criteria for informed consent.

**Deliberative interview with researcher**

I: I was at an eye specialist the other day and was given a form for taking part in a glaucoma screening. I found very little information on this form and asked: „Do you think I am at risk? Then I would consider it.“ It costs 40 Euros extra. That’s all the form said: it costs so and so much and I said: „Sorry, on that basis I am unable to make a decision.“

IP: No, no (affirming what the interviewer had said).

I: For me the information was lacking.

IP: Yes, exactly.

I: So sometimes I think there is also the problem of too little information…(researcher 3)

While the concern of information overload or incomprehensible legal or medical language on information sheets was raised by IPs in both interview styles, the point that insufficient information may also make informed consent difficult and is a questionable practice when combined with additional fees for medical interventions may not have arisen, if the interviewer had not shared her own experience.

Knowledge generation in conventional interviews also hinged on the way the questions were asked as these examples demonstrate:

**Conventional qualitative interview with NGO representative**

I: Can you think of another health policy? …. Or a policy that impacts on health?

IP: A policy that impacts on health? I am sure there are some. I can’t think of a practical example. Can you give me one?

I: Okay.. this one is a similar example: breast-cancer-screening which is offered every two years for women over 50. How do you view this policy?

IP: How do I view it? It’s a bit controversial, when you examine it more closely which most of the population may not be aware of.

I: If you were health minister, how would you go about this policy against the backdrop of debates about it?

IP: I would issue further studies and see that this is constantly evaluated to assess if the decision at the time was right, to see where one stands now, what the latest scientific results are in order to adjust the policy and not just to continue with it. (NGO 2)

**Conventional qualitative interview with researcher**

IP: In clinical studies it is clear, I think, it is a legal requirement that informed consent is necessary.

I: Informed consent by whom?

IP: Ah, by the patient.

I: By the patient… What about those conducting the study in clinical trials?

IP: Ah, in case those implementing the study are not the same as those responsible for the study? If doctors or surgeries are randomized to carry out an intervention in a health care study, then one would also need their consent. (researcher 3)

The theme of monitoring and evaluating a health policy may not have been mentioned if the interviewer had chosen a different and less controversial example of a health policy. With no further probing in the second example, the IP may not have raised the issue of consent by implementers.

Comparing knowledge generation across the two interview styles showed much overlap with some more depth and nuance being generated through the deliberative style. This was particularly evident for complex issues (health policy trials and health policies) and less evident for other issues (see Supplementary File 4). Additional points raised in deliberative interviews were, for example, personal, gender, religious and political implications for opt-in or opt-out clauses in health policies (e.g. not partaking in cancer-, syphilis- or pre-natal screening). Contradictory knowledge generation (e.g., implementer consent or decision-making power of local politicians for health policy trials) showed that exploring all options and arguments on which case-by-case decisions can be made may be more valuable than agreeing on one general course of action.

Knowledge generation for clinical trials and informed consent as a general theme did not show marked differences across interview styles – in fact some points were more nuanced in the conventional interviews. Some of these differences were undoubtedly due to the way the questions were asked (see Supplementary File 2). As we were keen to explore the IP’s position in the conventional interview and have a discussion in the deliberative interview the different approaches and questions asked led to different knowledge generation for the above themes.

## 4 Discussion

Our comparison of deliberative and conventional interview styles showed that deliberative interviews are more egalitarian in speaking time between interviewer and IP, that building on each other’s arguments in a deliberative interview was conducive to co-generating more nuanced knowledge for the main topics of interest and that rapport played an important role not just for conventional but also for deliberative interviews. A deliberative interview has been characterized as egalitarian in terms of the relationship between interviewer and IP and needing “less rapport” (Berner-Rodoreda et al., 2020). Trialing this new interview style, we perceive the need to alter certain assumptions about deliberative interviews.

Rapport plays a prominent role in conventional interviews and in particular in doxastic interviews which aim at understanding interviewees’ experiences or views and frequently use “friendship as a method” (Tillmann-Healy, 2003) for creating trust; rapport in deliberative interviews, by contrast, was assumed to play a subordinate role (Berner-Rodoreda et al., 2020). Yet, our findings show that in order to deliberate with someone and to contradict the interview partner without offending him or her requires good rapport, humor and a willingness to seriously engage with the other and the subject matter on the part of both, interviewer and interview partner. If the interview partner felt offended, this could easily end the deliberation and the interview. We therefore highlight the importance of rapport for the deliberative interview style and perhaps for epistemic interview styles in general. Rapport was important in the conventional interview as it is in all interviews, yet in comparing the two interview styles we would rate good rapport as a more essential requirement for conducting a deliberative interview.

The difficulty of stepping out of the interviewer or interviewee role should not be underestimated. Despite prior information of the dialogic nature of the deliberation, despite instructions that both interviewer and IP could interrogate each other’s ideas, our deliberative interviews were, for the most part, still conducted in the style of the interviewer asking questions, the IP responding, even if the interviewer was occasionally asked for her opinion or challenged by the IP. Scholars such as Bellah, Bourdieu, Brinkmann and Tanggaard have been critiqued for the discrepancy between their described ideal-type epistemic interview in terms of the co-construction of knowledge and the examples they presented which often resembled characteristics of doxastic-type interviews (Berner-Rodoreda et al., 2020); the same critique can be levelled against the chasm between theory and practice regarding the deliberative interview style. In theory it should or could be egalitarian (Berner-Rodoreda et al., 2020); in our experience it was more egalitarian in terms of speaking time than a conventional interview but we only reached the level of exchanging the role of interviewer and interview partner in few instances in the interview situation. Our experience showed the need for a more thorough methodological briefing prior to the interview in addition to the oral introduction of the methodology. For future deliberative interviews we propose to explore whether a short video on the methodology prior to the interview may enhance the egalitarian nature of a deliberative interview and inspire the interview partner to take on more of the interviewer’s role.

The assumption that the deliberative interview style is more aggressive and confrontational than other epistemic interview styles could not be confirmed; the degree of aggressiveness and confrontation largely depends on interview interactions. If one’s personal interview style is characterized by letting IPs finish their train of thought, then agreeing or disagreeing with the IP, and the IP agreeing or disagreeing with the interviewer’s points will likely mirror a normal conversation or dialogue. Our own experience showed that while challenges and disagreements occurred, the dialogue seemed more harmonious than anticipated. IP and interviewer provided positive feedback on the deliberative interview style: both enjoyed the interaction and engagement with each other’s arguments, being stimulated by the other’s thoughts and ideas and preferred it to a conventional interview style, even if the deliberative style initially felt strange.

Curato depicted how a captain she knew personally became the door-opener to interview detained military personnel in the Philippines (Curato, 2011). We also had the experience of IPs facilitating subsequent interviews with others who we may not have been able to interview otherwise because of their elevated positions and busy careers, yet who responded when greetings were passed on from one of their friends or former colleagues who had recommended them as an interview partner.

Various scholars have remarked and expounded on the implications of age, gender and social position on the interview situations (Curato, 2011; Kezar, 2003; Kvale, 2007; McDowell, 1998). As noted in Supplementary File 3, sharing the same gender, and age or interviewing a younger person of the same gender made the interview situation easier for both the deliberative and the conventional interview styles as it underscored commonality, perhaps even equality between interview partner and interviewer, or it provided the interviewer with slightly more authority as an older person. Being a middle-aged interviewer, the fact that half of the German interview partners were above the age of 50, appeared at first unproblematic; the experience of IPs holding monologues or veering off the topic seemed, however, more pronounced with some older and mainly male IPs who perhaps wanted to demonstrate the richness of their extensive work experience*,* which ultimately challenged a more substantive exchange. While the interviewer was treated with respect in all interviews, interviewing older highly educated interview partners in some cases left a feeling of ‘being the PhD student’ thus having to establish one’s equality by challenging the interview partner or making statements that showed one’s expertise - a strategy noted by others in interviewing elites (Kezar, 2003). Sharing a similar academic background or position, a similar personality and expertise regarding the issue to be discussed facilitated deliberation.

In both interview styles similar knowledge was generated – either through dialogue or through self-deliberation with the deliberative style producing slightly more knowledge, see Supplementary File 4, with the interviewer employing similar probes in both interview styles. Yet, the dialogic nature and more direct questions asked in the deliberative interview also facilitated ascertaining the IP’s position which was more difficult, at times impossible to determine through a conventional interview despite probing and playing devil’s advocate in both interview styles. The main difference in conducting the interviews lies in the provision of the interviewer’s opinion in the deliberative style leading to agreement, disagreement and mutual challenging. This approach also has a bearing on analysis as different perceptions on topics could either lead to exploring different facets of the topic or to a combined higher-level knowledge (see 3.1). The analysis of the theme thus happens within the interview situation (Berner-Rodoreda et al., 2020), and the interview process can be further analyzed by studying incidences of a change of opinion indicating the acceptance of a more compelling argument analogous to group deliberations (Abelson et al., 2013). In a conventional interview, by contrast, the analysis is mostly restricted to the ‘what’ rather than the ‘what and the ‘how’ (Gubrium & Holstein, 2003), whereas a thematic analysis is usually based on the answers by the interviewee alone and conducted after the interview is completed.

In terms of deliberation, personality and comparable knowledge levels seemed the most pertinent factors, as the deliberative interview style did not work well with people who talked profusely, nor did it work well with those who were unsure of their opinion or had not given the theme much thought. Deliberation worked best with the middle range between these two extremes: IPs who had an opinion on the issue, could verbalize it well and were prepared to listen and engage with the interviewer. We therefore propose further research not only on the role of gender, age, and academic/professional background but also on the role of personality for conducting deliberative interviews. Personality has been studied in the context of employment interview situations (Bourdage et al., 2020; Sears & Rowe, 2003); we propose to extend it to the context of research interviews as well.

While ‘interview’ may not be the most appropriate term for the deliberative exchange, it has proved difficult to find a suitable alternative nomenclature. ‘Deliberative dialogue’ is already used to denote group deliberations (Boyko et al., 2012; McCoy & Scully, 2002) rather than a dialogue of two people. Thus the term ‘inter-view’ in the sense of an exchange between two people (Kvale, 2007) has been maintained, yet other scholars may find a more appropriate terminology.

## 5 Conclusion

Our study has shown that deliberative interviews expedite clarity on the interview partner’s position, are conducive to co-generating knowledge and examining different facets of a complex theme within and, we believe, beyond public health. Irrespective of academic discipline, we would recommend employing this interview style for multifaceted social, health-related, political, environmental or economic challenges (e.g. pathways to halt climate change, designing a just political representation or a just taxation system, achieving greater evidence-based global learning in pandemics) which require mapping, deliberating and identifying promising solutions through a dialogue of informed individuals. The deliberative interview probably works best with knowledgeable individuals who are comfortable being challenged, who can argue for or against issues without undertaking personal attack or offense, and who remain open to changing their views in light of new information or insight. Whether the style works for highly emotional issues such as investigating the pros and cons of compulsory vaccinations may need further research. The deliberative interview style cannot be recommended for exploring and understanding the interview partner’s personal experience.

Our experience has shown that deliberative interviews are well accepted by interview partners, enjoyable to conduct and more egalitarian than conventional qualitative interviews. In order to maximize the added benefit of using the deliberative style over a conventional interview style, we confirm the importance of a thorough methodological briefing and similar knowledge levels on the subject matter (Berner-Rodoreda et al., 2020) and would add the willingness to meaningfully engage with the interviewer in a dialogue. Further research will have to determine the role of age, gender and academic or professional background for reaching an egalitarian relationship in this interview style and whether this style works best with compatible personalities.

## Ethical statement

This study was approved by the Heidelberg Ethics Commission in Germany (S-291/2018). All study participants were informed of the study orally and in writing. Voluntary written consent was requested and obtained from all study participants. Interview transcripts received pseudonyms to protect interview partners’ identity.

## Funding

This research was made possible through 10.13039/100010269Wellcome Trust grant No. 208766/Z/17/Z. There was no interference in the study design, data analysis or interpretation of results by the funder. Till Bärnighausen was supported by the 10.13039/100003579Alexander von Humboldt Foundation through the Alexander von Humboldt Professor award, funded by the Federal Ministry of Education and Research; the European Union; the 10.13039/100010269Wellcome Trust; and from 10.13039/100000071NICHD of NIH (R01-HD084233), NIA of NIH (P01-AG041710), 10.13039/100000060NIAID of NIH (R01-AI124389 and R01-AI112339) as well as FIC of NIH (D43-TW009775). Shannon A. McMahon was supported by the Olympia Morata Program of Heidelberg University.

## Declaration of interests

The authors declare that they have no known competing financial interests or personal relationships that could have appeared to influence the work reported in this paper.
