# Supplementary material for: “Thought provoking”, “interactive”, and “more like a peer talk”: Testing the deliberative interview style in Germany
Source: SSM Qual Res Health. 2021 Dec;1:None. doi: 10.1016/j.ssmqr.2021.100007 (PMC8688150; doi:10.1016/j.ssmqr.2021.100007)
Supplement: Multimedia component 1 [file mmc1.docx]

# **Deliberative Interview on Informed Consent**

Our interview topic is ‘informed consent’. Please read this background information sheet in advance of our interview as it provides important information on what we would like to discuss in the interview and is an integral part of a deliberative interview.

We will use the following working definition of informed consent: The permission that someone gives to be part of an experiment, surgery or treatment after being informed about all known benefits and risks.

## **Short History and Reference Documents for Informed Consent**

Informed consent became a central requirement in research ethics in the second half of the Twentieth Century. In relation to consent to participation in experiments, the Declaration of Helsinki stresses that ‘participation by individuals capable of giving informed consent as subjects in medical research must be voluntary’ (Article 25) and that each participant has the ‘right to refuse or withdraw consent’ (Article 26). Likewise, for medical interventions such as surgery or invasive procedures, informed consent is usually also sought from the patient after all risks and benefits have been presented and the patient has been made aware of his/her right to decline consent.

When it comes to health policy and to testing novel health policies for their population-wide effects (e.g. through cluster-randomized trials with a large population), the situation is complex. Individual level informed consent is only sometimes feasible. Consider fluoridating water supplies as a dental health measure. If this is implemented or even merely tested in some communities, individuals in these communities have practically no way to opt out (besides moving to a different community or buying lots of bottled water, either of which is very onerous).

The Council for International Organizations of Medical Sciences (CIOMS) in their ethical guidelines on health-related research involving humans holds up the importance of individual consent to medical research on human participants, but makes partial exceptions in cases where it is almost impossible to ask for informed consent such as in many cluster-randomized trials (CRTs) where ‘groups of individuals (clusters), communities, hospitals, or units of a health facility are randomized to different interventions’. In so-called individual-cluster trials, the individual has to accept the cluster randomization (e.g. communities or health centers being randomized) but can opt out individually, e.g. not being vaccinated in a cluster-randomized vaccine trial. By contrast, in cluster-cluster trials, the individual cannot usually opt out, e.g. when what is tested is an environmental change or a change to the health delivery system, the individual cannot avoid being affected by that change. In those cases, CIOMS says that “gatekeeper permission” (permission from community leaders, local health council) is necessary (CIOMS 2016).

In this interview we want to explore when in your opinion informed consent is needed and when it is not in health research, as well as what form any such consent should take.
