# Supplementary material for: “Thought provoking”, “interactive”, and “more like a peer talk”: Testing the deliberative interview style in Germany
Source: SSM Qual Res Health. 2021 Dec;1:None. doi: 10.1016/j.ssmqr.2021.100007 (PMC8688150; doi:10.1016/j.ssmqr.2021.100007)
Supplement: Multimedia component 2 [file mmc2.docx]

**IN DEPTH INTERVIEW GUIDES with Health Experts**

**Preliminary Remarks**

Our international research team, comprised of researchers from Heidelberg University (Germany), Harvard University (USA), BRAC University (Bangladesh), and Muhimbili University of Health and Allied Sciences (Tanzania), has been tasked to develop an international framework for ethics in health policy experiments. The aim of this framework is to improve governments’ ability to conduct such experiments ethically and effectively, and thus detect and eliminate ineffective health policies and invest scarce resources in more effective, efficient, and equitable approaches to healthcare delivery. In this pilot phase of the overall study, we will discuss health research with regard to the question of consent.

Our group is soliciting advice and comments about ethical aspects of health policy experiments from health experts like yourself. To carry out this consultation, we are also piloting a new interviewing technique called deliberative interviewing, and comparing it to conventional qualitative interviewing. You have been randomly assigned to a ­­­­­­­_______________________ interview. (qualitative/deliberative)

(Interviewer go to a) or b) based on random assignment)

1. Today, we will undertake a qualitative interview.

Speaking on behalf of everyone involved in this study, thank you for your willingness to help us think together about solutions to ethical challenges that arise whenever health policy is guided by especially-tailored experiments. Health Policy experiments test the impact of alternative models of health care and other policies that shape population health. Before we start, do you have any questions? (Interviewer go to Consent and Recording)

1. Today, we will undertake a deliberative interview (Interviewer continue)

This type of interview is a bit different than what you may be used to and will involve deliberation. You and I will debate, discuss and exchange ideas on questions that do not necessarily have a straightforward answer. Here are some interview parameters:

1. You have been provided background information on the theme that we are going to discuss.
2. In this dialogue, you are encouraged to voice your opinion and share your ideas and solutions.
3. I will describe some alternative ideas and solutions that could be considered. I will ask you to do the same, so that many facets of the issue are taken into account. You are free to provide additional observations and ideas at any time. You are warmly invited to disagree with me or to point out flaws in the approach that I put forward.

In some cases, I will respond to your remarks with follow-up questions. These will be useful in helping me gain clarity about your thinking. My hope is that by exploring complex issues from different perspectives, you and I can come to a higher level of thinking and generate unique, exciting and relevant insights to solve existing challenges.

**Consent and Recording**

Before we start the interview, can I ask you to read and, if you agree, sign the consent form? As the form states, your personal data will not be used in any publication, your participation is voluntary, and you have the right to withdraw at any time.

Thank you for signing the consent form. Our interview will probably last around 60 minutes. Do you have any questions before we begin?

Before we start the discussion, can I go over some personal data? (Interviewer to go to information about interviewee). As mentioned before, your personal data will only be looked at by the research team.

| **Indepth interview (IDI) cover page**  **Health Expert Interviews**  **(to be filled in by the interviewer)** | |
| --- | --- |
| **Name file as**  **Method (IDI)/Country first three letters/Type of interview (Conventional (C), Deliberative (D)/Institution (eg MED or NGO/ Interviewer Initials/ # of interview of the day /date day month year**  **(ETH- members of Ethics Commission or ethicists, MED- medical doctor; NGO- Health NGO, POL- policy makers, RES- researchers, UNI- students)**  **Example: IDI/GER/MED/C/AB/1/13.05.18** | |
| **Interview Date (Day-Month-Year)** |  |
| **City** |  |
| **Country** |  |
| **Institution/Professional Group** |  |
| **Type of Interview** |  |
| **IDI Interviewer** |  |
| **IDI Transcriber** |  |
| **IDI Translator** |  |
| **IDI duration (in minutes)** |  |
| **Have you verbally recorded the reference number?** |  |

| **Information about Interviewee**  (to be filled in **before *or* at the end of** the interview) | |
| --- | --- |
| Age |  |
| Gender |  |
| Present Position |  |
| Education level (name highest degree or educational level) |  |
| Profession |  |
| How long have you worked in the field of health? |  |

May I start the recording? *[Start recording]*

| **Conventional Qualitative Interview** |
| --- |
| 1. Thank you again for your willingness to participate in this interview. As mentioned before, this interview is about **informed consent.** In the interview I will present you with different scenarios and ask you, if you think someone should be consulted before a decision is made to test or introduce a new health policy and if so, how this should be done. |
| 1. Let us imagine a government that wants to improve health care for patients in hospitals and decides to increase the number of nurses by 10%. Since the health budget is limited, less money would be left for other medical interventions like upgrades of hospital machinery that in this case could not be fully realized. Still, the health ministry determines after thorough internal assessment that the population-health benefits of increasing the number of these professionals are greater than the harms to population health from being unable to afford other interventions. **Should anybody be asked for informed consent before this measure is taken?**   Follow up:   - **If so, who should be asked?** - **If not, why not?** |
| 1. Worldwide, sexually transmitted infections are a concern. If we take the example of syphilis, there are 5-6 million new infections p.a. worldwide. Early detection of syphilis facilitates both syphilis treatment and the prevention of syphilis infection of others. A government wants to check, if offering a blood test for syphilis to anyone who goes into a health facility, regardless of their health problem would detect and treat more cases of syphilis early.   The government randomizes all health facilities so that half of them offer the syphilis screening to whoever comes in for whatever treatment (though anyone can refuse the screening), and half offer syphilis for people who have syphilis-specific symptoms and/or ask for a test. The government in this case has not asked anyone for approval of the policy being tested, or the policy in each clinic (that’s determined by which arm the clinic was randomized for).  **How do you view this approach?**  Follow up:   - **An alternative would have been to ask local leaders/councils to decide if the health clinics in their area will take part in the trial or not. How would you view this alternative?** - **Which form of consent do you think is more appropriate?** |
| 1. So, imagine you are a citizen of this country and you live near a health clinic that takes part in this experiment. You have chest pain and go to the clinic. Upon arrival, you are being told by health personnel that everyone in this clinic is now offered a syphilis test, as the government wants to help everyone who tests positive for syphilis get treated immediately in order to lower the transmission of syphilis in the population. You are therefore being asked to do a test and to give written consent, and that you can decline at will. You are also being told that a new screening policy is being tested and that your personal results will not be shared with any persons except the investigators (who will receive them in anonymized form). The study’s conclusions will only report the cumulative number of cases, so nobody could identify you should you test positive. In this situation, **what would be your reaction to being asked if you wish to do a syphilis test?** |
| 1. Now let us imagine, as you entered the hospital you saw posters which explained the intention of the government to test everyone in the country in order to reduce transmission rates of syphilis as too many cases go undetected. **Would this information have changed your attitude towards accepting or declining informed consent?**   Follow-up:   - **What importance does information have for you in a trial like this?** |
| 1. Now say, after this trial was carried out for 18 months, data showed that more people were treated for syphilis in the health centres with (based on general surveys) fewer reported new cases of syphilis and no measurable effect on healthcare in other areas that were part of the active arm, so based on the evidence, the Government introduced this screening policy nationwide. So when we talk generally about health policies I mean, "decisions, plans, and actions that are undertaken to achieve specific healthcare goals within a society".   **Can you think of a health policy that has affected you personally?**  Follow up:   - **How was consent taken/asked for?** - **What would you have done, if you had been in a position to introduce this policy?** - **So, when you think generally of the fields of health policy and health policy research – in what circumstances do you think consent to the policy or the research is necessary?** - **If consent felt important: consent by whom?** - **If consent not important: why do you not think consent would be important?** |
| 1. Now we are moving from health policy and health policy research to a clinical trial: Imagine you are a middle aged man/woman who frequently suffers from the seasonal flu and is on sick leave for two weeks every winter. The doctor you go to has heard of a medical trial on a newly developed medicine against the seasonal flu which may help you, too, if it proves to be more effective than existing medication. The safety of this new medicine has already been tested and the new medication is compared against the standard medication which is offered to the control group.   The doctor asks you, if you are willing to take part in this trial and to give your written consent, if you agree. **What does ‘giving consent’ mean to you in this situation?**  Follow up:   - **How do you view ‘informed consent’ in this situation? (Would this be important or unimportant for you?)** - **Can you** **explain your position?**   (This is to find out, if IC is important or not important to the interviewee in a clinical trial situation and what he/she understands by informed consent in this situation) |
| 1. **What is your understanding of informed consent?** |
| 1. **Other than this interview can you recall when you were last asked to formally consent?**   Follow up:   - **How did you feel about this?** |
| 1. We are coming to the end of the interview: We have now looked at informed consent with regard to health related research,health related policies and clinical trials. **Can we come up with criteria when informed consent should be sought in health research, both: in clinical research as well as in health policy research?**   Follow up:   - **At what level should informed consent be sought?** |
| 1. **In what situations do you think a Government or research team does not need to ask for informed consent?**   Follow up:   - Should they do anything else instead? |
| 1. Thank you very much for sharing your ideas and concerns. **Is there anything else we did not cover in this interview which you would like to add?** |
| 1. Since this is a testing phase, we are also interested in any aspects you would change for future discussions. |
| 1. Once we get into the next research phase – **would you be willing to be interviewed again?** |
| 1. Thank you. **Is there anyone else you think we should talk to? Could you give us his/her details?** |
| 1. Thank you again for taking the time to share your experiences, thoughts and ideas. We very much appreciate it. I will stop the recording now. |
| **DELIBERATIVE INTERVIEW** |
| 1. Thank you again for your willingness to participate in this dialogue. As mentioned before, we will discuss and deliberate together, in what health context and with regard to which type of health research, some sort of consultation may or may not be necessary and what form it should take. We are conducting a dialogue, a conversation, so please feel free to challenge my views and ask me questions in the same way as I may challenge yours. This is not a one-way conversation. |
| 2. Imagine a government that wants to improve health care for patients in hospitals and decides to increase the number of nurses by 10%. Since the health budget is limited, less money would be left for other medical interventions like upgrades of hospital machinery that in this case could not be fully realized. Still, the health ministry determines after thorough internal assessment that the population-health benefits of increasing the number of these professionals are greater than the harms to population health from being unable to afford other interventions. **Should anybody be asked for informed consent before this measure is taken?**  Follow up:  **If so, who should be asked?**  **If not, why not?**  *Personal Notes for Interviewer/Suggestions for reactions:*  *I agree/disagree. I think it is up to the Government to introduce this. It is certainly a measure that is in the best interest of the patients****. Do you think there should have been a public debate about this?***  *It would be very unusual to ask for informed consent from anyone other than political decision makers here. It’s a governmental policy on health and those we elected to govern have come up with this measure hopefully by consulting with health facilities first so that this policy is based on evidence. After all, the government was democratically elected and in way that is all the consent that is needed for the government to act.*  *Let’s stay at a government level and look at another hypothetical example to discuss when consent of one form or another may or may not be necessary.* |
| 1. Worldwide, sexually transmitted infections are a concern. If we take the example of syphilis, there are 5-6 million new infections p.a. worldwide. Early detection of syphilis facilitates both syphilis treatment and the prevention of syphilis infection of others. A government wants to check, if offering a blood test for syphilis to anyone who goes into a health facility, regardless of their health problem would detect and treat more cases of syphilis early.   The government randomizes all health facilities so that half of them offer the syphilis screening to whoever comes in for whatever treatment (though anyone can refuse the screening), and half offer syphilis for people who have syphilis-specific symptoms and/or ask for a test. The government in this case has not asked anyone for approval of the policy being tested, or the policy in each clinic (that’s determined by which arm the clinic was randomized for). **How do you view this approach?**  Follow up:  An alternative would have been to ask **local leaders/councils to decide if the health clinics in their area will take part in the tria**l or not**. How would you view this alternative**?  *Personal Notes for Interviewer/Suggestions for reactions:*  ***Arguments against the alternative approach of asking local leaders/councils:*** *I personally do not really think it would be necessary to go through the local leaders. I think, if health facilities are randomized in the whole country, there is no bias in the distribution of where the screening is offered and where it is not offered. From that point of view I regard the Govt randomization as better science than doing it through local leaders and councils, as their personal interest or apprehension about this trial might confound the results. It is not randomly distributed – e.g. local leaders may refuse the intervention precisely at precincts that (as the leaders suspect) are those where the intervention is likely to fail, as people may be embarrassed to be tested for syphilis and therefore not make use of the health facilities. What’s important to me is that a trial is conducted before a new policy is introduced.* ***What is your view on this?***  ***Arguments for the alternative approach of asking local leaders/councils, if IP is against it****: At the same time, having local leaders engaged might also encourage people to go for testing and it is generally always good to consult with people beforehand. This way, if and when some individuals are offended by the offer of testing for syphilis they could be answered that in a sense their representatives have offered their consent to this. while representatives’ consent is not precisely individuals’ consent, the latter is impracticable, and representative consent is the closest second best.*  ***How would you view consulting the entire population, like holding a referendum on the randomization approach?*** |
| 1. So, imagine you are a citizen of this country and you live near a health clinic that takes part in this experiment. You have chest pain and go to the clinic. Upon arrival, you are being told by health personnel that everyone in this clinic is now offered a syphilis test, as the government wants to help everyone who tests positive for syphilis get treated immediately in order to lower the transmission of syphilis in the population. You are therefore being asked to do a test and to give written consent, and that you can decline at will. You are also being told that a new screening policy is being tested and that your personal results will not be shared with any persons except the investigators (who will receive them in anonymized form). The study’s conclusions will only report the cumulative number of cases, so nobody could identify you should you test positive. **In this situation, what would be your reaction to being asked if you wish to do a syphilis test?**   *Personal Notes for Interviewer/Suggestions for reactions: I agree/I disagree:*  ***For*** *this approach: under these conditions, there is very little risk, if any, from being tested. And you can always opt-out if you want to. Not to offer the test would catch fewer cases of syphilis early or at least prevent the possibility of checking if more cases are caught early by offering the screening to all.*  ***Against*** *this approach: If there was no information beforehand, it is a bit of a shock to be asked to do an syphilis* *test when you come into the clinic with a totally different health problem and are unprepared for this. Also, depending on the way the health personnel introduced this test-all approach to you, you may feel that the health personnel thinks you are at elevated risk for syphilis* *and therefore may feel stigmatized. If individuals are offended, they might not return to the clinic, and warn others not to come. This may affect population health in other areas, or even reduce early detection of syphilis (inasmuch as some who avoid coming have syphilis and don’t want it detected). Even though this is a mere trial, to spread distrust in clinics in this way risks instilling distrust that may affect the precinct long after the trial is over.* |
| 1. Now let us imagine, as you entered the hospital you saw **posters** which explained the intention of the government to test everyone in the country in order to reduce transmission rates of syphilis as too many cases go undetected. Would this information have changed your attitude towards accepting or declining informed consent?   Follow-up: What **importance does information** have for you in this situation?  *Personal Notes for Interviewer/Suggestions for reactions: I agree/disagree.*  *If think reading the information before I see a doctor or nurse would make a huge difference in feeling they are not just asking me - this is a general screening policy so I would feel this would help in accepting this new measure.*  *But on the other hand, the poster lend this issue salience. So some people reading this may think, this was the last time I am going to this health facility and might find out what facilities are not part of the trial in which case we have not gained much as this person may have syphilis and then pass it on to others. So, in my view information beforehand could have a positive or a negative effect****. How do you view this?*** |
| 1. Now say, after this trial was carried out for 18 months, data showed that more people were treated for syphilis in the health centres (based on general surveys) with fewer reported new cases of syphilis and no measurable negative effect on healthcare in other areas that were part of the active arm, so based on the evidence, the Government introduced this screening policy nationwide. So when we talk generally about health policies I mean, "**decisions, plans, and actions that are undertaken to achieve specific healthcare goals within a society".** We had the example earlier where a government health policy (increasing the number of nurses) was not tested beforehand.   **So, when you think generally of the fields of health policy and health policy research – in what circumstances do you think consent to the policy or the research is necessary?**  *Personal Notes for Interviewer/Suggestions for reactions:*  *I think it depends on the type of policy or research. If people feel that there are certain real or perceived risks, then I think it would be best to consult people beforehand either through gate-keepers or directly. Since most policy research is conducted in cluster randomized trials such as the fluoridation of water supplies in communities or the release of genetically modified mosquitoes to reduce the transmission of infectious diseases like dengue or zika, I think we need to see, how we can get maximum engagement of the population but also ensure that we do not jeopardize advances in public health.* ***How do you see this?*** |
| 1. Now we are moving from health policy and health policy research to a clinical trial. Imagine you are a middle aged woman who frequently suffers from the seasonal flu and is on sick leave for two weeks every winter. The doctor you go to asks you, if you would like to be part of a medical trial on a newly developed medicine against the seasonal flu. The safety of this new medicine has already been tested and the new medication is compared against the standard medication which is offered to the control group.   **What is your position on whether or not the doctor should ask you for informed consent?**  *Personal Notes for Interviewer/Suggestions for reactions:*  *If IC seen as crucial:* ***Why do you think it’s so important to consent – the safety of this medication has already been tested and if you end up in the control group, you are getting the standard of care medication.***  *If IC not seen as important:* ***What about the risks of taking a new medication that might have serious side-effects? If consent is not sought, could the doctor not do what he/she likes with the patients and put them on a trial without them being aware of it?*** |
| 1. If you recall the background sheet we sent you. We gave the rough definition of informed consent as ‘as **‘the permission someone gives to be part of an experiment, trial, surgery or treatment after being informed about all the known benefits and risks.’** Now that we have discussed informed consent in more detail, **is there some clarification or qualification you might want to add?** |
| 1. Other than this interview can you recall when you were last asked to formally consent?   *Personal Notes for Interviewer/Suggestions for reactions: Share your own thoughts/experience with informed consent.* |
| 1. So, we have now looked at health policies, health policy experiments and clinical research as well as situations where personal consent was asked for. Can we come up with criteria **when informed consent should be sought** in health research, both: in clinical research as well as in health policy research?   *Personal Notes for Interviewer/Suggestions for reactions:*  *I think that in line with all the ethical frameworks, the Helsinki Declaration, CIOMS, etc. - in any cases where there are real or perceived risks to study participants, informed consent should be sought but perhaps we should look at all scenarios.*  *Clinical Trials: I think for most clinical trials this is a given as there is usually a certain risk to study participants.*  *Health policy research is often done in Cluster Randomized Trials. So, in many cases, individual consent is almost impossible and the individual cannot opt out*. ***Is community consent or gatekeeper consent acceptable?***  *To me it would depend on who is consenting for others. If it is a community board with a cross-section of the community I would feel better than an individual who could easily be influenced one way or another.* ***What do you think?***  ***At what level should informed consent be sought?*** (only ask, if not already answered in the discussion) |
| 1. In what situations do you think a government or research team does not need not ask for either individual consent or community consent or consent through a referendum and simply make decisions on its own?   Follow up:  A government does not usually consult people before introducing a new policy. **Should they do that in the context of research – whether it is a clinical study or a study of this or that health policy?**  *Personal Notes for Interviewer/Suggestions for reactions:*  *My view is that there needs to be clear scientific evidence for the benefit of a health policy before it is introduced or not, or very little risk to the population. A case in point is the prohibition to smoke in restaurants. I don’t see that this needs the consent of everyone. Smokers would certainly not agree but it is a good health measure. Breast cancer screening and other screening programs are ones I can use but that I can also decline so I do not see that I need to be asked beforehand.* ***Can you think of examples where people should be asked for consent before a policy is introduced?***  *We also looked at the quality improvement measure of increasing the number of nurses. Often they only refer to a small change to service delivery. I believe most of them do not need informed consent.* ***What’s your view?***  *I think if nobody – neither patients nor health care workers – is put at much risk through the intervention or through disclosure of private information, one could waive consent but I would define the risks not only in medical terms but also in social terms such as increased stigma****. Do you agree or do you see things differently?***  ***Should researchers or the government do anything else instead in terms of permission or information before a trial is conducted or a policy introduced?***  *I’d say in most cases people should be informed if it does not interfere with the study as such.* |
| 12. Great.Thank you so much for your participation and your reasoning. This has been very helpful. Is there any aspect we did not cover that you wanted to add to the discussion? |
| 13. Well, we may not have come up with one model for when informed consent might apply and when it may not apply but we have certainly produced a lot of insightful ideas. Now since this is a new interview style, can I ask you how you felt participating in this type of interview? |
| 14. What appealed to you? |
| 15. What did you find difficult or burdensome? Your feedback is valuable for us as this is a new interview style. |
| 16. Since this is a testing phase, we are also interested in any aspects you would change for future discussions. Please share your insights. |
| 17. Thank you for your suggestions. Is there anyone else you think we should talk to? Could you give us his/her details? |
| 18. Once we go into the next phase of research – would you be willing to be interviewed again?  Thank you very much for your time and for sharing your thoughts and ideas.  We very much appreciate it. I will stop the recording now. |

| **Interviewer comments / notes**  (to be filled in by the interviewer ***after*** the interview) | |
| --- | --- |
| Reflexive Comments:  How well do you think this interview went?  What do you feel worked, what didn’t work?  What may have to be done differently in the future?  Please make any other notes you feel are relevant or important to add here. | |
| Was this interview interrupted?  Tick one:   [ ] Yes       [__] No | If yes, who interrupted? ____________________  Number of times interview was interrupted?  [Insert number]  ________ |
