# Supplementary material for: “Thought provoking”, “interactive”, and “more like a peer talk”: Testing the deliberative interview style in Germany
Source: SSM Qual Res Health. 2021 Dec;1:None. doi: 10.1016/j.ssmqr.2021.100007 (PMC8688150; doi:10.1016/j.ssmqr.2021.100007)
Supplement: Multimedia component 3 [file mmc3.docx]

Supplementary File 3 Comparison of conventional and deliberative interview styles, Germany

*Legend: Numbers in brackets refer to the number of times an issue was mentioned*

| **Characteristics** | **Conventional** (10 interviews) | **Deliberative** (10 interviews) |
| --- | --- | --- |
| **Interview Engagement** | | |
| Purpose of interview | Generating knowledge on informed consent across various contexts and study types by focusing on reasoning of interview partner (IP). | Co-generating knowledge on informed consent across various contexts and study types through dialogue. |
| Interview relationship, information flow and speaking time (based on NVivo coding) | IP was interviewed as expert and listened to. Interviewer mostly let IP finish talking before probing or asking further questions.  Information flow was one-directional (from IP to Interviewer). IP’s average speaking time was 67% (ranging from 58% to 78%) compared to interviewer’s average speaking time of 28% (ranging from 18% to 36%). | While relationship between interviewer and IP should be equal and roles reversable, it proved difficult to break out of interviewer-interviewee mold. Interviewer mostly let IP finish talking before probing, challenging or asking further questions. Information flow went both ways. IP’s average speaking time was 52% (ranging from 34% to 69%) compared to interviewer’s average speaking time of 48% (ranging from 28% and 60%) |
| Sharing of interviewer knowledge and experience | No – except for answering questions of clarification on the examples, introducing alternative examples and occasionally health policy examples for IP to discuss. A question regarding the interviewer’s opinion on the matter was reverted back to IP. | Yes – sharing own experience of informed consent and own position on questions; when IP had not thought or felt unsure about issues, interviewer tended to share more. Sometimes interviewer introduced additional issues, examples of health policies or study types that would not need informed consent. |
| Interview rapport | Moderately important – since interview contained few personal questions, rapport did not seem to play an overarching role. | Important – if IP did not feel comfortable, it would have been even more difficult to have a true exchange. |
| Role of Interviewer | Neutral. Mainly probing and asking for clarification. In some interviews interviewer challenged and highlighted inconsistencies. | Fully engaged in dialogue - reacting, agreeing, disagreeing, sharing, challenging, yet dialogue mainly based on interviewer asking questions. Agreements exceeded disagreements; yet interviewer disagreed with and challenged IP more than vice versa. |
| Role of Interview Partner (IP) | Answering questions. In 9/10 interviews, IP also asked questions– mostly clarifications regarding the question they were asked or the example they were given. | Engaged in dialogue, yet IP still largely answering questions. In all interviews IPs asked questions, yet mostly about contents or rhetorical. In 3/10 interviews IP asked interviewer for opinion. In all interviews IP agreed with interviewer, in 6/10 interviews IP disagreed or challenged interviewer. |
| IP changing his/her mind | IPs argued by themselves the pros and cons and what the better way forward might be. Sometimes when challenged about inconsistencies, IP might change opinion. | IP changing his/her mind occurred at times when interviewer put a different argument forward. IP also tended to change opinion when an issue had higher personal priority or when s/he regarded the scenario as higher risk. |
| Consensus reached | Consensus could not be reached as interviewer did not provide own opinion. | Consensus often reached between IP and interviewer: health policy trials and health policies should be evidence-based; health policies only need expert opinion and political authorization; referendums can be manipulated and should be used sparingly; gate-keepers should be democratically legitimized; no need for prior individual consent if opt-out is possible. |
| Manner of challenging and reception to challenging | Interviewer challenged in general terms “Some people may say/argue..” “If I may play devil’s advocate..” or pointed out in- consistencies: “You mentioned before that… now you seem to say..” Since IPs regarded interview topic as generally challenging, additional questions seem to make little difference to IP. | Interviewer provided own opinion and mentioned other considerations, challenged by pointing out inconsistencies, questioned why IP held that opinion or played devil’s advocate. Interviewer holding a different opinion, challenging or bringing in other aspects did not seem problematic for IP. |
| How IP felt about interview | When asked, IPs felt that questions were fine, but difficult to answer.  Specific Feedback (some IPs mentioned more than one point):  made me reflect deeper; stimulating; hadn’t thought about this before (3), interesting field or study (3), good to conduct study on this issue (2), issue and ethical dimension is greater and more complex than thought of before (3), felt increasingly uncertain of own opinion during course of interview (1) | All IPs were asked how they felt about the interview style and provided the following feedback (some IPs mentioned more than one point):  Very pleasant (7), new/surprising/unusual, feels more like a peer talk (2), interesting and exciting, one gets new impulses (1), positive (1), more relaxed as both share opinion (1), better than answering set questions on one’s own (4), ideas develop through dialogue (2), one gets feedback on own opinion and can reflect (2), more thought-provoking than other interview styles (1), works well if one is not an expert (1), needs good rapport as it feels like sharing something personal (1), may not work well if great discrepancy in knowledge (1), may also work in groups (1) |
| How interviewer felt about the interview | General feeling of having to hold back, especially when conducting deliberative interviews before. Good rapport in all interviews.  Pleasure to conduct interview with knowledgeable, experienced and engaged IPs (irrespective of gender or age), who could express ideas well and added own examples. It was often challenging to find out what IPs’ positions were. For some interviews it may have been easier to establish IP’s position through a deliberative interview style. Some IPs had a tendency to veer off topic and lose themselves in details of examples and needed to be brought back to questions. Conducting interviews with IPs of same gender and age or younger or with IPs of similar academic background appeared easier. For generating knowledge, IP’s reflected thoughts on the matter played a crucial role irrespective of IP’s gender, age or academic background. | Generally feeling more relaxed as interviewer could fully participate. In most interviews it felt like a discussion/dialogue. Good rapport in all interviews,  Pleasure to conduct interview with knowledgeable, experienced and engaged IPs (irrespective of gender or age), who could express ideas well and added own examples. When IP veered off topic a lot, mentioned too many details or talked profusely, it felt more like a conventional interview. Shorter answers by IP led to a snappier dialogue. When IP was not sure about own position, interviewer shared more and IP tended to agree with interviewer. Interviewer’s style of letting IP generally finish train of thought may have established more rapport, yet may have also made interview less confrontational. Interviews were easy to conduct with IPs of same gender and younger or same age and with IPs with similar professional work, background or personality irrespective of gender. Willingness to engage with each other’s ideas seemed most crucial for co-generating knowledge. |
