# Supplementary material for: “Thought provoking”, “interactive”, and “more like a peer talk”: Testing the deliberative interview style in Germany
Source: SSM Qual Res Health. 2021 Dec;1:None. doi: 10.1016/j.ssmqr.2021.100007 (PMC8688150; doi:10.1016/j.ssmqr.2021.100007)
Supplement: Multimedia component 4 [file mmc4.docx]

Supplementary File 4 Knowledge Generation on Informed Consent

| **Conventional interview** | **Mentioned in both interview styles** | **Deliberative interview** |
| --- | --- | --- |
| **What considerations or aspects are important in relation to informed consent in Health Policies (HPs) ?** | | |
| **Other important considerations**   - A policy decision should be preceded by public debate or an ethical discourse in society | **Who should be involved in decision-making?**   - Politicians, parliament - Independent expert committee composed of relevant representatives (patient reps, associations of doctors, hospitals, insurers); lobbyists should not be included - National Ethics Council/Committee - Implementers should be consulted - Asking entire population (= conducting referendum) is not suitable for HPs.   **Important Features**   - HP needs to be based on scientific evidence - The common good is greater than the individual right to consent (e.g. compulsory vaccination) - Policy can have opt-in and opt-out features   **Other important considerations**   - Survey can be conducted prior to introducing HP - Information to population is important - No IC prior to policy introduction necessary, if opt-out exists - Regular M+E of policies on scientific basis | **Who should be involved in decision-making?**   - Experts with differing opinions have to be involved so that all aspects will be considered. - Politicians make decision about HP, even if experts hold different opinion - Expert opinion may be opposed by majority population views, yet does not call for decision by populace.   **Important Features**   - HP needs to protect vulnerable groups - Policy should allow for opt-out at individual level for personal/religious reasons. - Affected need thorough background on opt-in/out options and their consequences. If opting-out or not opting-in has negative consequences for individual, individual should not be penalized for decision.   **Other important considerations**   - HP should be tested before being introduced - Economic considerations for policies (is it worth the expenditure?) |
| **What considerations or aspects are important in relation to informed consent in Health Policy Trials (HPTs)?** | | |
| **Other important considerations**   - Local democratic bodies need to be convinced with good arguments and evidence - Some people will not benefit personally but should not be harmed through the intervention | **Who should be involved in decision-making?**   - Independent experts need to be involved - Ethics Commission or National Ethics Council have to approve HPT - Involvement of consent-givers depends on urgency of HPT and on risks.   - If risk low, consent through democratic structures.   - If risk very low, could be introduced as policy.   - If risk higher: IC through referendum. If referendum not possible: survey or community dialogue - Implementers should be asked to consent before randomization to achieve higher compliance. - Implementers should not be asked to consent to avoid bias. - Involvement of community reps should not be based on one individual, but groups (e.g. community boards)   **Important Features**   - HPE needs to be epidemiologically justified and of low risk - Information important (before, during, after) and could be more important or alternative to consent - Randomization is ok, if equipoise can be guaranteed. - Implementers need to be aware of stigma implications of IC. - Participation should be voluntary, if type of HPT permits   **Other important considerations**   - Alternative study designs should be explored before conducting an HPT based on randomization - Country context is important | **Who should be involved in decision-making?**   - Involve experts with pro and con-arguments. - Local politicians/counsellors should not approve HPT as they lack scientific know-how - Local politicians/counsellors can make decision on HPT being implemented in their area after being well-informed about matter by experts.   **Important Features**   - Cluster trials constitute grey area for informed consent.   **Other important considerations**   - Information about HPT could lead to spill-over effect in a randomized trial - HPE (RCT) needs good follow-up and tracing to measure effects |
| **What considerations or aspects are important for informed consent in Clinical Trials?** | | |
| **Who should be involved in decision-making?**   - May need consent from implementers if it is health services research   **Important Features**   - Information should include background, aim of study, right to leave study and explain randomization and responsibilities to participant.   **Other important considerations**   - Risk of implementer pressuring some patients to take part in study | **Who should be involved in decision-making?**   - Participants - Ethics commission needs to approve trial.   **Important Features**   - IC very important, as product not yet approved - Participants need to self-determine, if they want to participate - Needs information on benefits, also benefits for the common good, risks/side-effects, and data handling - No need for informed consent if comparing approved standard therapies   **Other important considerations**   - Needs trust in implementer and trust in science (willingness to support medical progress) | **Important Features**   - Information should include termination criteria of study - Clinical trials need consent as industry involved through medical products and personal data is being collected - It would destroy trust in doctor/implementer and raise suspicion of vested interest, if participant not asked for IC   **Other important considerations**   - Implementer/Doctor should inform about alternatives to taking part in this study |
| **What considerations or aspects are important for informed consent in general?** | | |
| **Who should be involved in decision-making?**   - If minors can be affected negatively, IC should be given by health personnel   **Important Features**   - Trust in doctor makes IC less important; if medical confidentiality guaranteed, no need for consent   Other important considerations   - an alternative to informed consent could be ‘implied consent’ such as in   Norwegian model (general patient consent)   - IC is like a contract: consent not needed if people feel responsible for each other | **Who should be involved in decision-making?**   - Consent to medical interventions by patient - Consent by representative if child cannot consent   **Important Features**   - Expression of self-determination and human dignity. Necessary for measures/research affecting individual if invasive, have potential to cause psychological or physical harm or use personal data. - Needs to be voluntary and conscious decision by individual who should have time and room for decision and know all benefits and risks - Can be given orally or in writing - Sometimes overdone with long information sheets or lengthy consent procedures - Information needs to be framed in easy to understand language. - No prior consent needed from individuals for: emergency situations and health crises, health policies, for anonymized data, if opt-out exists or risk is minimal | **Who should be involved in decision-making?**   - Groups of people who need a representative to consent as they cannot provide consent themselves: people with dementia, people with psychological problems and children.   **Important Features**   - Too little information may lead to ‘selling’ services/interventions to client which are unnecessary. - Individual IC needs to be asked objectively, without bias. - Agreement with definition provided in background briefing: “the permission someone gives to be part of an experiment, trial, surgery or treatment after being informed about all the known benefits and risks” - Definition does not cover consent in cluster studies through gatekeepers or representatives or for changing environments. Some felt that ‘written’ should be added or that all known benefits could be reduced to all relevant benefits and risks for that particular person. - No prior consent needed for observation studies, register studies or quality improvement studies |
